# Senolytic drugs relieve pain by reducing peripheral nociceptive signaling without modifying joint tissue damage in spontaneous osteoarthritis

**DOI:** 10.18632/aging.204204

**Published:** 2022-08-10

**Authors:** Tae-Hwan Gil, Haiyan Zheng, Hyo Gyeong Lee, Ji-Won Shin, Sun Wook Hwang, Ki-Mo Jang, Ok Hee Jeon

**Affiliations:** 1 Department of Biomedical Sciences, College of Medicine, Korea University, Seoul 02841, Republic of Korea; 2 Department of Physiology, College of Medicine, Korea University, Seoul 02841, Republic of Korea; 3 Department of Orthopaedic Surgery, Anam Hospital, College of Medicine, Korea University, Seoul 02841, Republic of Korea

**Keywords:** senescence, spontaneous osteoarthritis, pain, senolytics, nociceptive pathways

## Abstract

Aging is a risk factor for the development of osteoarthritis (OA), a progressive joint disease leading to cartilage damage, pain, and loss of function. In a mouse model of OA, senolytic drugs to selectively clear senescent cells (SnCs) that accumulate with injury or aging yielded a chondroprotective effect; however, this therapeutic benefit was limited in aged mice. Due to inconsistency between cartilage destruction and pain-associated symptoms, we studied the therapeutic effect of senolytics on joint pain in spontaneous OA. We orally treated 21- and 22-month old mice with an ABT263 and Dasatinib and Quercetin (D+Q) drug combination. Selective elimination of the SnCs that accumulated in the articular cartilage and synovium by these two drugs did not alter cartilage degeneration and abnormal bone changes during spontaneous OA progression. Treatment reduced thermal and mechanical hyperalgesia associated with OA and peripheral sensitization through decreased expression of axon guidance proteins (nerve growth factor NGF/TrkA) and nociceptive neuron (calcitonin gene-related peptide, CGRP) projection to the synovium, subchondral bone marrow, and dorsal root ganglion, and knee joint angiogenesis. Selective removal of the SnCs from *in vitro* cultures of synovial cells from human OA patients also decreased expression of senescent markers, axonal growth-promoting factors, such as NGF, and angiogenesis markers. We suggest that systemic administration of ABT263 and D+Q is an exciting therapeutic approach to age-related OA pain.

## INTRODUCTION

Osteoarthritis (OA) is a common degenerative joint disease, particularly in individuals over the age of 60 [1]. Although younger populations are at risk of developing OA after joint trauma, aging is a crucial risk factor for OA. OA is mainly characterized by joint structural changes, such as cartilage degradation, synovial inflammation, subchondral bone alterations, and persistent joint pain, leading to impaired mobility and reduced quality of life [2–4]. The extent to which structural changes in OA contributes to presence or severity of pain has been controversial. There is a clinical discordance in OA between pain sensation levels and arthroscopic and histological evidence of articular cartilage degeneration [5, 6].

Cellular senescence is broadly characterized by a permanent proliferative arrest, apoptotic resistance, and the secretion of extracellular matrix-degrading and pro-inflammatory molecules termed senescence-associated secretory phenotypes (SASP), which can induce structural and functional changes in the surrounding cells and tissues [4]. Studies of human tissues and mouse models have shown an increased incidence of senescent cells (SnCs) during aging and joint tissue degeneration [7–9]. For example, significantly increased senescent chondrocytes and synovial fibroblasts are found in human articular cartilage and synovium derived from OA patients and a post-traumatic mice model [8]. Subsequent removal of these SnCs with senolytics ameliorates post-traumatic OA progression by reducing injury-related pain and inflammatory marker expression and increasing cartilage development in young animals. In contrast, when a similar treatment was administered to aged animals, injury-associated pain and inflammatory markers were reduced, but cartilage degradation was not. Consistent with this result, long-term treatment with a combination of Dasatinib (D), a Src/tyrosine kinase inhibitor, and Quercetin (Q), a natural flavonoid that binds to BCL-2, which has been used extensively as a senolytic, did not inhibit the degradation of articular cartilage in a spontaneous animal model of OA [10]. Thus, it must be clarified if removal of SnCs has a therapeutic effect in a spontaneous OA, particularly joint pain.

Both clinical and preclinical research suggest that OA-related pain is induced by increased nociceptive input from the joint through alterations in pain signaling pathways in the central and peripheral nervous system [11, 12]. For example, activation of nociceptive neurons in the dorsal root ganglion (DRG), which is located in the intervertebral foramina in the peripheral nervous system, through nerve growth factor (NGF) to activate nociceptive neurons by binding tropomyosin receptor kinase A (TrkA), chemokine (C-C motif) ligand 2 (CCL2), tumor necrosis factor (TNF), and Netrin-1 correlates with OA-related pain [13–16]. Moreover, these axon guidance proteins induce nociceptive neuron projection locally in multiple joint tissues, including synovium and subchondral bone, leading to an exaggerated pain response by noxious mechanical (hyperalgesia) or innocuous stimuli (allodynia) [11, 12]. These peripheral sensitizations have been accessed by behavioral testing in OA mice models to indicate OA-related pain. Many disease-modifying therapies for OA pain have been developed and tested in clinical and preclinical studies, including NGF antibody [17], TrkA inhibitor (AR786) [18], and protein kinase C-δ (PKCδ) overexpression [19]. Currently, it is unknown whether senolytic drugs affect the degree of innervation of sensory nerve fibers in the synovium and subchondral bone and if there are subsequent changes to nociceptive signaling pathways, like CGRP and NGF/TrkA, to alleviate OA-related joint pain.

Here, we investigated the therapeutic potential of senolytics against a spontaneously developed OA. Using 21 and 22- month-old mice, we analyzed the effects of two senolytic drugs (ABT263 and D+Q) on structural alterations (including articular cartilage and subchondral bone degeneration and synovitis) and pain in knee joints. We further analyzed pain-related sensory innervation and axonal growth-promoting factors that stimulate neuronal sprouting in the joints and DRG and knee joint angiogenesis to address putative nociceptive mechanisms by which senolytic treatment reduces OA pain. *In vitro* cultures of synovial cells derived from human OA patients were used to confirm the applicability of our findings to human OA disease.

## RESULTS

### Senolytic drugs (ABT263 and D+Q) eliminate senescent cells in the joints of a spontaneous OA model

**Figure 1:**
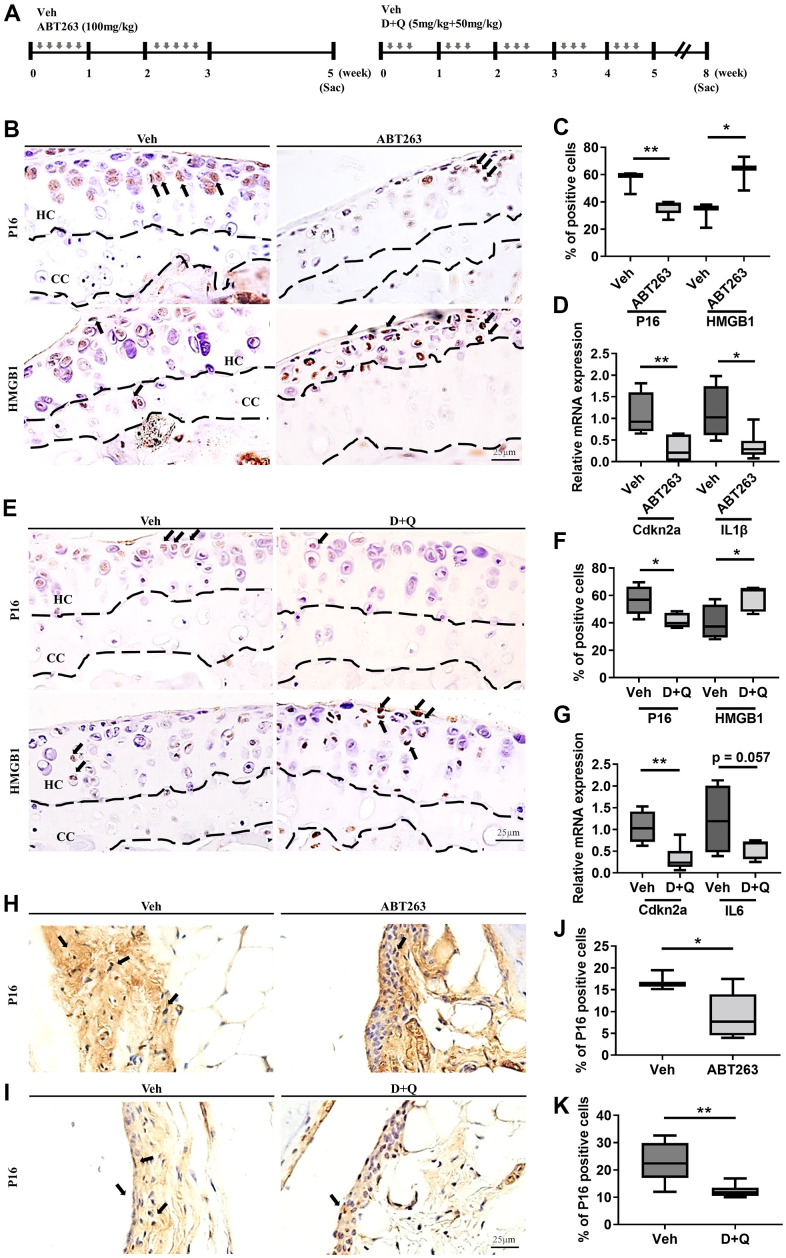
**ABT263 and D+Q deplete senescent cells in articular joints of aged mice.** (**A**) Timeline of the ABT263 and Dasatinib (D) + Quercetin (Q) treated aged mouse model of spontaneous OA. We orally administered ABT263 at 100 mg/kg or Vehicle (Veh) to 21 to 22-month-old mice five times biweekly for 4 weeks. The 21 to 22-month-old mice were given oral D + Q (5 mg/kg Dasatinib plus 50 mg/kg Quercetin) or Veh three times every week for 5 weeks. (**B**) Representative immunostaining images of p16 and HMGB1 + cells (arrows) in the articular cartilage of the ABT263 or Veh-treated aged mice. HC, hyaline cartilage; CC, calcified cartilage. (**C**) Quantification of p16 + SnCs and non-SnCs positive for nuclear HMGB1 in the articular cartilage (*n* = 3 mice for Veh, *n* = 5 mice for ABT263). (**D**) mRNA expression levels of *Cdkn2a* and *IL1β* are validated by qRT-PCR analysis in the aged mice administered ABT263 or Veh (*n* = 4 for Veh, *n* = 7 for ABT263). (**E**) Representative immunostaining for p16 and HMGB1 positive cells (arrows) in the articular cartilage of D+Q or Veh treated aged mice. (**F**) Quantification of p16 and HMGB1 + cells in the articular cartilage (*n* = 5 per group). (**G**) Quantification of mRNA levels for *Cdkn2a* and *IL6* by qRT-PCR in the joints of D+Q or Veh-treated aged mice (*n* = 4 for Veh, *n* = 7 for D+Q). Representative images of immunostaining for p16 in the synovium of (**H**) ABT263 or Veh-treated or (**I**) D+Q or Veh-treated in the articular knee joint of the aged mice. Percentage of p16+ cells in the synovial membrane of (**J**) ABT263 or Veh-treated (*n* = 3 for Veh, *n* = 6 for ABT263) or (**K**) D+Q or Veh-treated mice (*n* = 5 for Veh, *n* = 6 for D+Q). Whisker plots represent the 10^th^ and 90^th^ percentiles, and the line corresponds to the median. * p < 0.05, ** p < 0.01; a two-tailed Student’s t-test (unpaired). Scale bars, 25 μm.

It has been previously reported that SnCs accumulate in the articular cartilage and synovium after joint injury, and selective removal of SnCs attenuated the progression of post-traumatic OA and related pain in young animals [8]. Beyond UBX0101, we pursued the potential of other senolytics such as ABT263 and a Dasatinib (D) and Quercetin (Q) combination (D+Q) as senescence-associated therapeutic candidates to treat age-associated spontaneous OA. We first examined whether 20 to 21-month-old mice might be a suitable animal model of spontaneous OA. Safranin-O staining revealed that these mice exhibited age-related cartilage degeneration in comparison to those of 10-weeks-old mice, as evidenced by faint proteoglycan staining and significantly higher Osteoarthritis Research Society International (OARSI) scores (Supplementary Figure 1A, 1B). To evaluate if ABT263 and D+Q administration depletes SnCs in the articular cartilage and synovium of mice in a spontaneous OA model, we treated 21 to 22-month-old wild-type C57BL/6J mice with five times biweekly oral gavages of ABT263 or weekly oral gavage of a D+Q (Figure 1A) and evaluated the abundance of key senescence markers. Immunohistochemistry for p16^INK4a^, a biomarker of cellular senescence [20], was down-regulated in the tibial articular cartilage of the ABT263-treated aged mice compared to the Vehicle (Veh)-treated controls. Also, ABT263 treatment increased the number of non-SnCs positive nuclear high mobility group box 1 (HMGB1), an extracellular alarmin whose nucleus expression occurs before SASPs production in SnCs [21] in the articular cartilage of aged mice (Figure 1B, 1C). Selective SnCs clearance was also confirmed by reduced mRNA expression of *p16^INK4a^* (or cyclin-dependent kinase inhibitor 2A; *Cdkn2a*) and *Interleukin 1β (IL1β)* after ABT263 treatment (Figure 1D).

Similar to ABT263 treatment, D+Q decreased immunostaining for p16^INK4a^-positive senescent chondrocytes and increased the number of non-SnCs with nuclear HMGB1 (Figure 1E, 1F) and *Cdkn2a* and *IL6* (*p* = 0.057) mRNA expression in the articular cartilage of aged joints (Figure 1G). ABT263 or D+Q treated aged mice, when compared to mice without treatment, had a smaller number of p16^INK4a^-expressing SnCs in the synovium (Figure 1H–1K). These results suggest that treatment of ABT263 and D+Q targets and reduces the number of SnCs in the articular cartilage and synovium in a spontaneous OA progression.

### Elimination of senescent cells by senolytic drugs (ABT263 and D+Q) alleviates joint pain but does not reduce structural changes in the joint tissues during spontaneous OA progression

**Figure 2:**
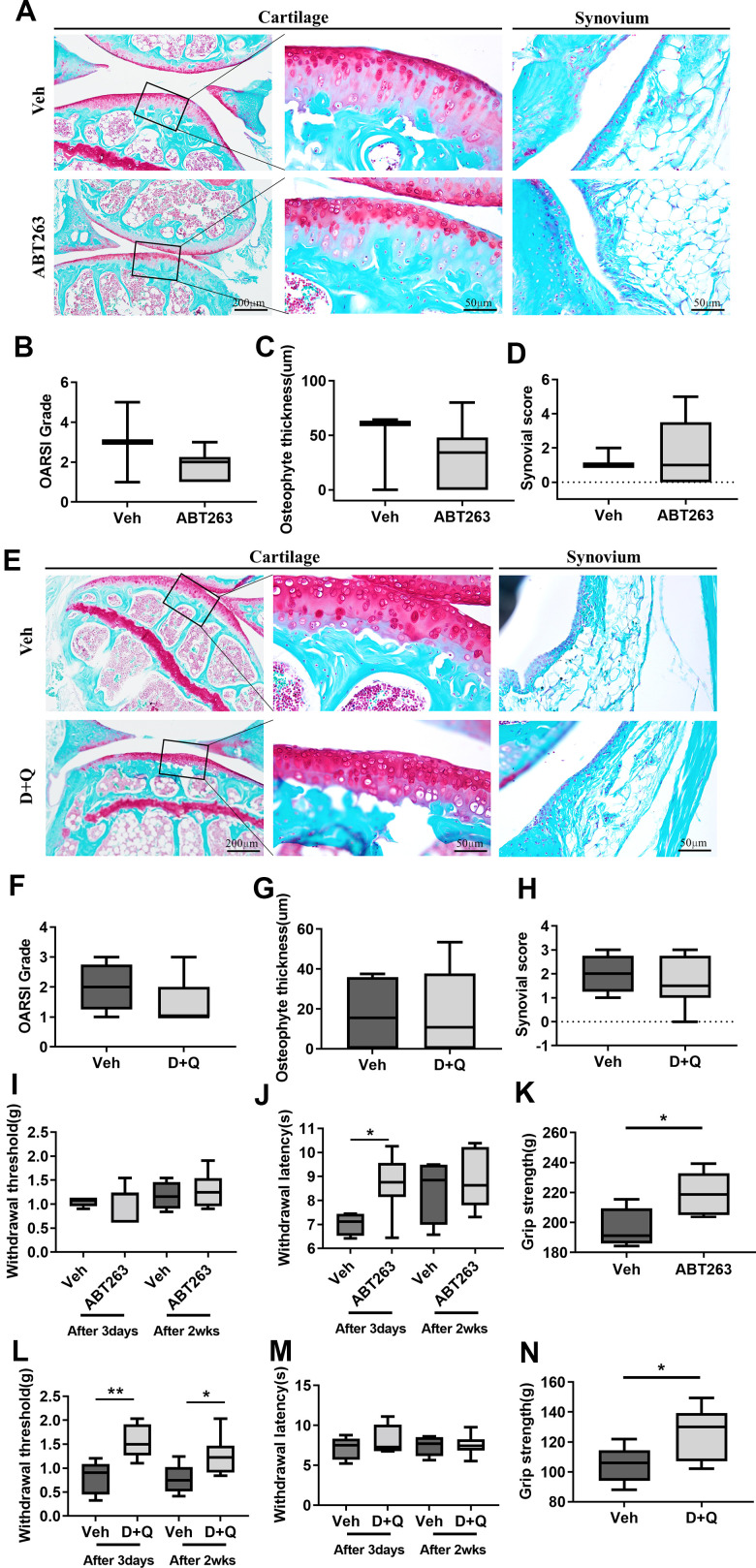
**ABT263 and D+Q alleviate joint pain but do not affect cartilage degeneration during spontaneous OA progression.** (**A**) Representative images of Safranin-O in the articular cartilage (left panel), higher magnification of the boxed regions (middle panel), and Safranin-O images of the synovium (right panel) of ABT263 and Veh-treated 21 to 22-month-old mice joints. (**B**) OARSI grade assessed by Safranin-O staining images in the medial tibial plateau, (**C**) osteophyte thickness of tibial articular cartilage, and (**D**) Krenn-synovitis score of ABT263 or Veh-treated aged mice (*n* = 3 mice for Veh, *n* = 6 mice for ABT263). (**E**) Representative images of Safranin-O staining in D+Q or Veh treated 21 to 22-month-old mice. Quantitative analysis of (**F**) OARSI grade, (**G**) osteophyte length, and (**H**) Krenn-synovitis score in the D+Q or Veh-treated aged mice (*n* = 4 for Veh, *n* = 8 for D+Q). (**I**) Von-Frey test to assess mechanical allodynia of the hind paws, (**J**) thermal hyperalgesia by Hargreaves apparatus, and (**K**) grip strength on day 3 and week 2 after the last dose of ABT263 treatment (*n* = 4 for Veh, *n* = 7 for ABT263). (**L**) Von-Frey test, (**M**) thermal hyperalgesia by Hargreaves apparatus, and (**N**) grip strength on day 3 and week 2 after the last dose of D+Q treatment (*n* = 5 for Veh, *n* = 8 for ABT263). Whisker plots represent the 10^th^ and 90^th^ percentiles, and the line corresponds to the median. * p < 0.05, ** p < 0.01; Unpaired Student’s t-test. Scale bars are shown in each image.

We then explored the consequences of selective removal of naturally occurring SnCs by senolytics (ABT263 and D+Q) on OA development in aged mice. In the articular joints of ABT263-treated mice, elimination of SnCs did not reduce age-related cartilage degeneration, abnormal osteophyte formation, and synovitis in comparison to Veh-treated aged mice. There was no change in Safranin-O proteoglycan staining, osteophyte lengths, and normalized OARSI [22] and Krenn-synovitis scores [23] (Figure 2A–2D). D+Q treatment also did not affect spontaneous cartilage destruction, as manifested by fibrillation or fissures, osteophyte development, and synovitis in a spontaneous OA mouse model (Figure 2E–2H). These correlate with no changes in the thickness and area of cartilage, calcified cartilage, and subchondral bone as demonstrated by histomorphometric measurements of the aged joints after both treatments (Supplementary Table 1).

Pain is a key, disabling complaint of patients suffering from OA. We examined whether ABT263 or D+Q decreased pain by performing the Von Frey filament assay, Hargreaves test [24, 25] and grip strength (Supplementary Figure 2). ABT-treated joints showed no change in mechanical hypersensitivity, measured by paw withdrawal threshold (Figure 2I), but significantly decreased thermal hyperalgesia, as measured by the Hargreaves test (Figure 2J) compared with Veh-treated joints on day 3 after ABT263 administration. This mitigative effect of ABT263 disappeared in week 2 after ABT263 treatment. We next tested whether ABT263 treatment impaired grip strength [26], which is often related to symptomatic OA pain. Grip strength was significantly increased in ABT263-treated mice compared with animals treated with Veh (Figure 2K). However, D+Q treated joints showed significantly lower mechanical allodynia, as measured by the Von Frey test 2 weeks and 3 days after the treatment. No difference in thermal hyperalgesia, measured by the Hargreaves test, or grip strength was noted when this group was compared to mice treated with Veh (Figure 2L–2N). Collectively, elimination of SnCs by ABT263 and D+Q in the aged joints alleviated OA-like pain but did not affect age-related joint destruction and synovitis during spontaneous OA progression.

### Spontaneously developed OA pain amelioration by senolytic treatment is associated with decreased nociceptive neuro-fiber projection in the synovium

**Figure 3:**
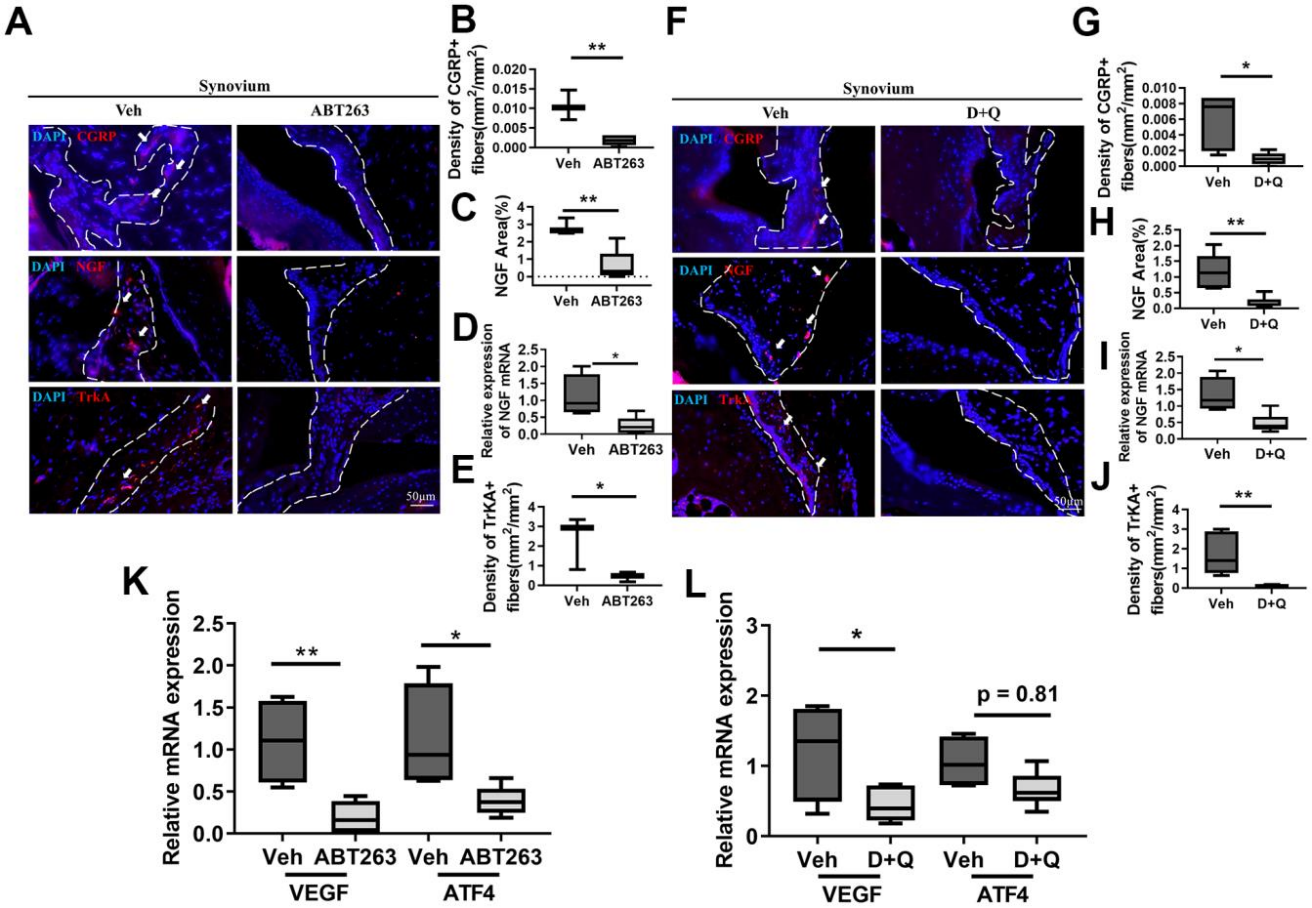
**Senolytics treatment (ABT263 and D+Q) decreases the projection of nociceptive neuro-fiber in the synovium in spontaneous OA in aged mice.** (**A**) Representative immunofluorescence images for CGRP, NGF, and TrkA in the synovium of 21 to 22-month-old mice were administered ABT263 or Veh (synovium indicated by dashed lines). Quantification of (**B**) density of CGRP+ nerve fibers (*n* = 3 mice for Veh, *n* = 5 mice for ABT263), (**C**) NGF positive area (*n* = 3 for Veh, *n* = 5 for ABT263), (**D**) relative mRNA levels of *NGF* in the joints (*n* = 4 for Veh, *n* = 7 for ABT263), and (**E**) density of TrkA+ nerve fibers in the mouse joint synovium (positive for each marker indicated by arrows) (*n* = 3 for Veh, *n* = 5 for ABT263). (**F**) Images of immunostaining of CGRP+ neuro-fibers, NGF expressions, and TrkA+ fibers. Quantitative analysis of the (**G**) density of CGRP+ sensory nerve fibers (*n* = 5 per group) and (**H**) NGF positive area in the synovium of aged mice administered D+Q (*n* = 5 for Veh, *n* = 7 for D+Q). (**I**) Relative mRNA levels of *NGF* in the joints (*n* = 4 for Veh, *n* = 6 for D+Q). (**J**) Quantitative analysis of the density of TrkA+ nerve fibers in the synovium of aged mice after D+Q administration (*n* = 5 for Veh, *n* = 7 for D+Q). Relative mRNA levels of *VEGF* and *ATF4* in the joint of (**K**) ABT263 or Veh (*n* = 4 for Veh, *n* = 7 for ABT263) and (**L**) D+Q or Veh-treated older mice (*n* = 4 for Veh, *n* = 6 for D+Q). Whisker plots represent the 10^th^ and 90^th^ percentiles, and the line corresponds to the median. * p < 0.05, ** p < 0.01; Unpaired Student’s t-test. Scale bars, 50 μm.

The pronounced induction of peripheral nerve sprouting during OA hyperalgesia development led us to investigate the synovium neurons and axonal growth-promoting factors that stimulate neuronal sprouting. Immunostaining of calcitonin gene-related protein (CGRP), a potent vasodilator that causes pain sensitization [15], showed that projections of CGRP positive nociceptive nerve fibers and their density nerve endings in the synovium decreased in ABT263-treated aged mice compared with Veh-treated controls (Figure 3A, 3B). SnC clearance by ABT263 significantly decreased immunostaining for synovial expression of the NGF protein for axon guidance in the synovium and the mRNA levels of *NGF* in aged joints (Figure 3C, 3D). TrkA, an NGF receptor [27–30], was reduced after ABT263 treatment in a spontaneous OA mouse model (Figure 3E). In support with the findings of these mechanistic research, D+Q treatment also reduced immunostaining for CGRP+ peptidergic sensory nerve fibers (Figure 3F, 3G) and lowered protein and mRNA expression of NGF with TrkA+ neuron projection into synovium compared with Veh-treated aged mice (Figure 3H–3J).

We also observed that CGRP+ neuron axon projection in subchondral bone remarkably decreased with ABT263 and D+Q treatment (Supplementary Figure 3A–3C). A recent study demonstrated that abnormal subchondral bone remodeling by osteoclasts can induce nociceptive nerve innervation and OA pain [15]. We then tested whether these inhibitory effects on nociceptive neurons in the joint tissues were derived from the aberrant subchondral bone formation by osteoclasts. Tartrate-resistant acid phosphatase (TRAP)+ osteoclasts were not altered by ABT263 nor D+Q treatment in a mouse model of spontaneous OA.

OA in animals causes pathological angiogenic activation that mimics that in patients with arthritic joint pain and increases local pain receptors that contribute to structural damage and pain [31–33]. We analyzed the mRNA levels of vascular endothelial growth factor (VEGF) and activating transcription factor 4 (ATF4), a transcriptional factor that regulates angiogenesis, in the joints of ABT263 or D+Q treated aged mice. ABT263 and D+Q treatment decreased the mRNA levels of *VEGF* and *ATF4* compared with Veh-treated controls (Figure 3K, 3L). Pathological angiogenic activation and vascular development are inextricably linked, particularly in arthritic joint tissue damage and pain [33–35]. Thus, we further performed stain CD31, endothelial marker, and found that ABT263 and D+Q treatment reduced the development of vasculature in the synovium in the spontaneous OA (Supplementary Figure 4A–4C). Thus, invasion of nociceptive neurons, active axonal growth in the joints, and joint angiogenesis were decreased by both senolytic agents during a spontaneous OA progression.

### Senolytics (ABT263 and D+Q) eliminate senescent fibroblast-like synovial cells from human OA tissue and decrease axon guidance and angiogenic factors

**Figure 4:**
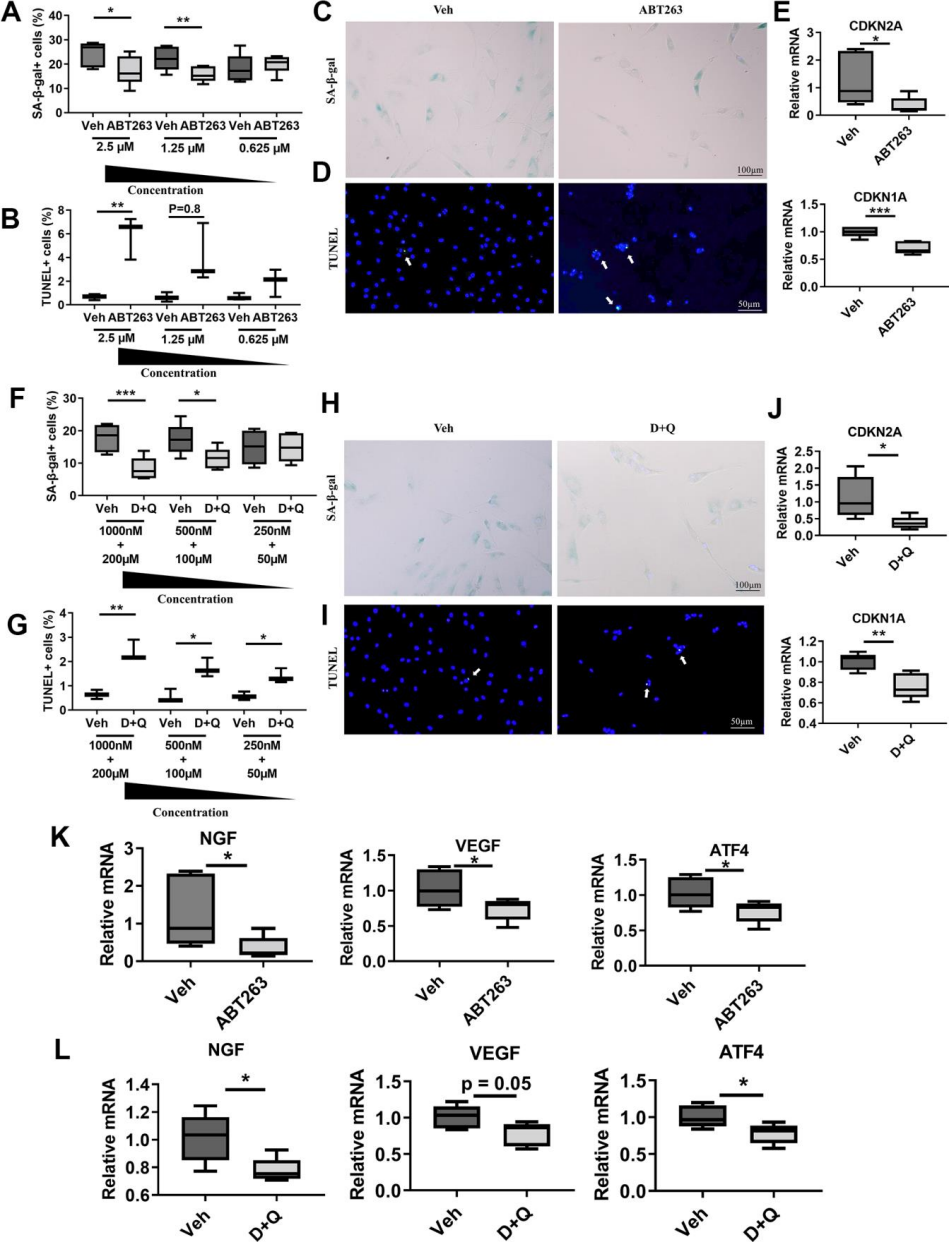
**Senolytics (ABT263 and D+Q) decrease the senescence phenotype population and axon guidance and angiogenic factors in fibroblast-like synovial cells from human OA tissue.** Quantification of (**A**) SA-β-gal+ (*n* = 6 per group) and (**B**) TUNEL+ cells in human OA fibroblast-like synovial cells treated with ABT263 (2.5 μM, 1.25 μM, and 0.625 μM) (*n* = 3 per group). Representative images of (**C**) SA-β-gal staining and (**D**) TUNEL staining of synovial cells treated with 1.25 μM of ABT263 or Veh for 3 days. (**E**) Relative mRNA expression levels of *CDKN2A* and *CDKN1A* in the synovial cells treated with ABT263 (1.25 μM) or Veh (*n* = 5 for Veh, *n* = 6 for ABT263). Percentage of (**F**) SA-β-gal + (*n* = 6 per group) and (**G**) TUNEL+ cells (*n* = 3 per group) in D+Q (D 1000 nM, 500 nM, 250 nM + Q 200 μM, 100 μM, or 50 μM). Representative images of (**H**) SA-β-gal staining and (**I**) TUNEL staining of human OA synovial cells treated with D+Q (D 500 nM + Q 100 μM) for 3 days. (**J**) Relative mRNA expression levels of *CDKN2A* and *CDKN1A* in the synovial cells treated with D+Q (D 500 nM + Q 100 μM) or Veh (*n* = 5 per group). Relative mRNA expression levels of *NGF, VEGF,* and *ATF4* in the synovial cells treated with (**K**) ABT263 (1.25 μM), Veh (*n* = 5 for Veh, *n* = 6 for ABT263), (**L**) D+Q (D 500 nM + Q 100 μM), or Veh (*n* = 5 for Veh, *n* = 6 for D+Q). Whisker plots represent the 10^th^ and 90^th^ percentiles, and the line corresponds to the median. Two independent experiments were performed. * p < 0.05, ** p < 0.01, *** p < 0.001; Unpaired Student’s t-test. Scale bars are shown in each image.

The relevance of SnCs clearance in clinical OA pain was evaluated in *in vitro* cultures from patients undergoing total knee replacement. We examined whether ABT263 and D+Q decrease the senescence phenotype population in fibroblast-like synovial cells from human OA tissue and consequently impact the expression of axon guidance and angiogenesis-related markers, such as NGF, VEGF, and ATF4. A preliminary screening showed that 1.25 μM is the minimum concentration of ABT263 required to remove senescent synovial cells, as determined by senescence-associated β-galactosidase (SA-β-gal) and TUNEL staining (Figure 4A, 4B). To further understand the effects of ABT263 on OA, human OA synovial cells were exposed to 1.25 μM of ABT263 for 3 days. ABT263 decreased SA-β-gal+ cells (Figure 4C) and increased apoptosis of synovial cells (Figure 4D) and decreased mRNA levels of *CDKN2A* and *CDKN1A* (Figure 4E).

A preliminary screening showed that 500 nM of Dasatinib + 100 μM of Quercetin were minimum dose to remove senescent fibroblasts like synovial cells (Figure 4F, 4G). Similar to ABT263, 500 nM of Dasatinib + 100 μM of Quercetin treatment for 1 day 1) decreased SA-β-gal positive human OA synovial cells (Figure 4H), 2) elevated apoptotic cell death as measured by a TUNEL assay (Figure 4I), and 3) decreased *CDKN2A* and *CDKN1A* gene expression (Figure 4J), indicating selective clearance of the senescent synovial cells in human OA tissues. Moreover, ABT263 and D+Q treatment also downregulated *NGF* gene expression and the angiogenesis marker *VEGF* and *ATF4* in human OA synovial cells compared with Veh-treated controls (Figure 4K, 4L). These data suggest that clearance of senescent synovial cells in human OA tissues by ABT263 and D+Q treatment may reduce age-associated OA pain through axonal growth-promoting factors, like NGF, and angiogenesis.

### Administration of senolytics suppresses activation of nociceptive neurons in DRG in spontaneous OA

**Figure 5:**
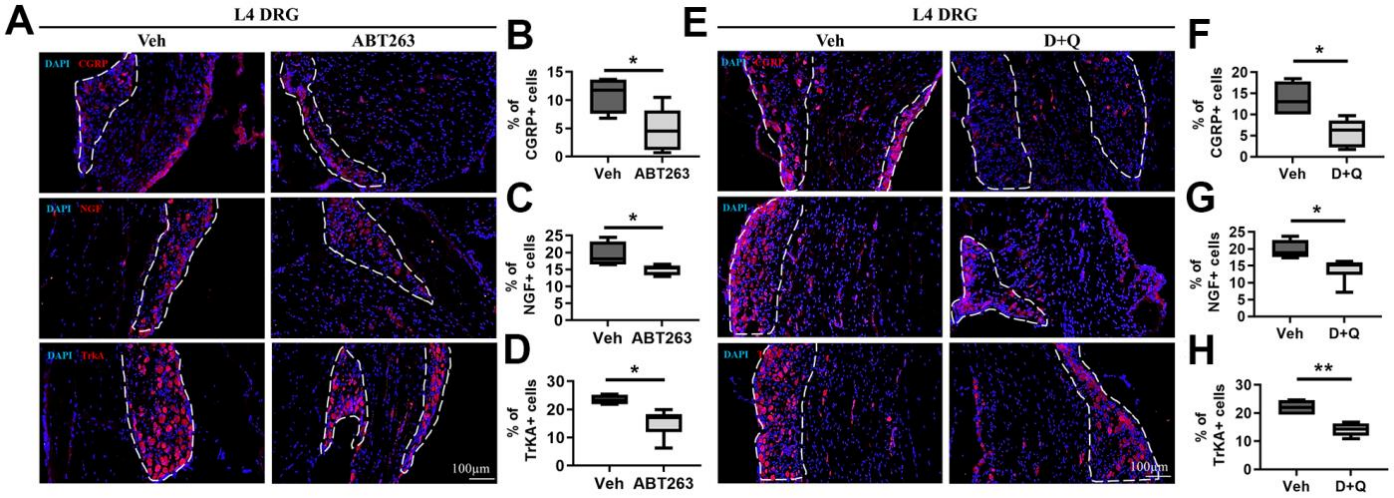
**ABT263 and D+Q decreased pain-related activation of DRG neurons in age-associated OA mice.** (**A**) Representative immunofluorescence images for CGRP, NGF, and TrkA of L4 DRG in 21 to 22-month-old mice administered by ABT263 or Veh. White dots indicate a DRG region. (**B**–**D**) Quantitative analysis of CGRP+ (*n* = 4 mice for Veh, *n* = 6 mice for ABT263), NGF+ (*n* = 4 for Veh, *n* = 5 for ABT263), and TrkA+ neurons (*n* = 4 for Veh, *n* = 6 for ABT263) in L4 DRG of ABT263 or Veh-treated aged mice. (**E**) Immunofluorescence analysis of CGRP +, NGF+, and TrkA+ neurons (red) in L4 DRG of D+Q or Veh-treated aged mice. (**F**–**H**) Quantification of CGRP+, NGF+, and TrkA+ neurons in L4 DRG of D+Q or Veh-treated aged mice (*n* = 4 for Veh, *n* = 6 for D+Q). Whisker plots represent the 10^th^ and 90^th^ percentiles, and the line corresponds to the median. Unpaired Student t-test was used for statistical analysis. * p < 0.05, ** p < 0.01; Unpaired Student’s t-test. Scale bars, 100 μm.

The development of OA pain is linked to neuronal plasticity, which involves changes in gene or protein expression in DRG sensory neurons and conveys pain signals. We thus examined whether ABT263 and D+Q alter the peripheral pain signaling pathways CGRP and NGF/TrkA as key components of OA pain [36]. In DRG neurons, CGRP, NGF, and TrkA expression levels were notably decreased by ABT263 administration as demonstrated by immunofluorescence staining (Figure 5A–5D). We also found that D+Q treatment strikingly suppressed CGRP + neuro-fibers and NGF and TrkA expression levels in L4 DRG compared with that of Veh-treated mice (Figure 5F–5H). Collectively, ABT263 and D+Q treatment alleviated the DRG neuron activation-associated pain phenotype, which correlated with pain-related behavioral changes during age-associated OA development.

## DISCUSSION

Despite the increased medical costs associated with joint OA in older persons, there are few pharmacological agents available, with NSAID or steroids used for inflammation and pain relief with limited therapeutic pathological joint changes [37]. The role of senescence in age (defined as primary OA) and trauma-related (defined as secondary OA) degenerative joint disease has recently garnered significant attention. Our previous study revealed that the elimination of SnCs by senolytic drug (UBX0101) reduced the development of post-traumatic OA and related pain and created a prochondrogenic environment in young animals. However, when the same treatment was intra-articularly administered to aged animals with joint injury, OA-associated pain and inflammatory markers were reduced, but cartilage degradation was unchanged [8], implicating that other senolytics or dosing methods might be required to attenuate OA in aged mice joints.

Here, we evaluated the therapeutic effects of systemic treatment of two senolytic drugs – ABT263 and D+Q, which have demonstrated promising results in a variety of tissues – in spontaneous OA. We revealed that senolytic drugs (ABT263 and D+Q) could target senescence in aged mouse joints and subsequently alleviate joint pain, but do not mitigate articular cartilage degeneration during spontaneous OA progression. Additionally, we revealed that this amelioration of spontaneous OA-related pain is due to inhibition of nociceptive neuron invasion, active axonal growth into the aged joints, knee joint angiogenesis, and nociceptive neuron activation in the DRG. Our findings suggest that senolytics may be useful in relieving age-associated pain in spontaneous OA progression.

While many senolytic agents have been studied, ABT263 (Navitoclax) is the first class targeting the anti-apoptotic BCL-2 and BCL-xL proteins that rebuild bone marrow components, such as mesenchymal progenitors and hematopoietic precursors in aged animals. This could help with tissue repair in aged joints [38]. D+Q is another class of senolytic that interferes with SnCs pro-survival pathways [39]. Both drugs reduce the senescence burden in multiple tissues and attenuate age-related pathologies.

Previous human tissues and mouse model studies demonstrate an increased incidence of SnCs during aging and with joint trauma [7, 8]. Despite this causative relationship between SnCs and OA pathology, senolytic treatment has not been used in a mouse model of spontaneous OA regarding pain or joint structural changes. We treated 21 to 22-month-old wild-type C57BL/6J mice with evident age-related cartilage degeneration with orally administered ABT263 or D+Q. We then analyzed the cartilage and subchondral bone degradation and synovial inflammation in age-related arthritic joints. Systemically administered drugs are usually absorbed into the joint cavity with a filtrate of plasma as called synovial fluid from synovial capillary. Many studies have been reported that drugs administrated by oral gavage distribute or even accumulate into synovial fluid [40, 41], suggesting that senolytics could be entered to the joint cavity with oral treatment.

Our data demonstrated that systemic administration of ABT263 and D+Q did not rescue the age-related cartilage degradation and synovitis despite a decreasing senescence phenotype in the cartilage and synovium in mice with spontaneous OA. This result is consistent with data that suggests that long-term D+Q treatment starting in 6, 14, and 18-month-old mice (without joint injury) did not attenuate cartilage degradation [10]. Systemic removal of SnCs positive for p16^INK4a^, an important biomarker for cellular senescence, in the cartilage tissue did not affect OA progression in a surgically induced OA mouse model (with joint injury) [42]. This may be due to a decrease in the ability of articular chondrocytes to proliferate and synthesize which is necessary to intervene in the degenerative process, or the senescence of cartilage tissues is not the sole factor for age-related cartilage degeneration. SnCs present in multiple tissues (such as subchondral bone, infrapatellar fat pad, etc.) in the knee joints may be simultaneously targeted to improve the therapeutic efficacy of senolytic drugs.

A rat model of surgically-induced OA showed increased sensitivity to mechanical and thermal stimulation as well as CGRP and NGF+ sensory neuron innervation into the synovium when compared to mice without joint pain [36, 43]. We tested ABT263 and D+Q treatment’s ability to relieve pain through nociceptive signaling pathway alterations (CGRP and NGF/TrkA) during peripheral sensitization in a spontaneous OA mice model. Our results showed that systemic administration of ABT263 and D+Q to aged mice reduced OA-associated pain behavior. The decreased expression of 1) CGRP+ neuro-fibers to innervate into the synovium, 2) NGF proteins for axon guidance, reducing TrkA, the NGF receptor in nociceptive neuron, in the synovium, and 3) activation of CGRP and NGF/TrkA + nociceptive neurons in DRG, might underlie the peripheral pain sensitization. This may be reflected in the decreased mechanical and thermal hyperalgesia by both senolytic treatments in aged mice. This result is in line with previous findings that clearance of senescent-like neuronal cells in DRG, through a senolytic (ABT263) intervention and p16-3MR transgenic mouse model, reduced mechanical and thermal hyperalgesia in a mouse model of cisplatin-induced peripheral neuropathy [44].

We also investigated whether sensory nerve innervation in subchondral bone is associated with DRG neuron projection following decreased age-related OA pain noted in behavior tests in spontaneous OA joints. ABT263 has potential adverse effects on osteoprogenitor function and bone loss [45]. Furthermore, osteoclasts are activated during abnormal bone remodeling and Netrin-1, an axon guidance molecule, is produced by osteoclast-induced nociceptive neuron innervation into the subchondral bone in mediating OA pain [15, 46]. Our study revealed that, although neuro-fiber projection was decreased in the subchondral bone marrow of aged mouse joints after ABT263 and D+Q treatment, there was no change in the osteoclast population. Aberrant bone formation (osteophyte and subchondral bone volume) after ABT263 and D+Q treatment was not observed compared to vehicle-treated aged mice. These results indicate that a decrease in the projection of CGRP+ nociceptive neurons into the subchondral bone and pain hypersensitivity might not be derived from osteoclast activity.

It is yet to be determined whether SnCs in other organs like lymphoid tissues, soluble molecules in the circulation, or a combination of these factors are responsible for the observed alleviation of arthritic joint pain by orally treated senolytics. Our previous study demonstrated that systemic administration of ABT263 reduced a type 17 immune response in the draining inguinal lymph node and even articular compartment in young and aged mice with post-traumatic OA [47]. This previous study could explain that the systemic treatment of senolytics may cause numerous systemic changes including the immune system, to improve the pain response and may also regulate cartilage tissue degeneration in the spontaneous OA. As senolytics sufficiently control age-related OA pain, a better understanding of the molecular mechanism by which cellular senescence contributes to OA-related pain may potentially help develop more effective pharmacological agents for OA treatment.

In summary, we determined that the systemic administration of ABT263 and D+Q in aged mice alleviated OA-associated pain in a spontaneous mouse model of OA. The potential benefits of these two senolytic drugs treatments include inhibition of nociceptive neuronal invasion, reduced axonal growth in aged joints and knee joint angiogenesis, and suppression of nociceptive neurons in the DRG of aged mice. This study suggests that the senolytic drugs ABT263 and D+Q may be an exciting therapeutic approach to age-related OA pain. Our work also reinforces the need for more effective pharmacological senolytic agents to address the challenges of limited aged joint tissue regeneration, enabling the development of disease-modifying OA drugs.

## MATERIALS AND METHODS

### Animals and drug treatments

We obtained 13-month-old male C57BL/6J mice from the aging research facility of the Korea Basic Science Institute (Gwangju, Republic of Korea), and kept them until they reached 22 months of age. We administered the 22-month-old male C57BL/6J mice with the vehicle, ABT263, or Dasatinib plus Quercetin (D+Q) diluted in 10% ethanol, 30% polyethylene glycol 400, and 60% Phosal 50 PG (Lipoid, #NC0130871, Germany). ABT263 (APExBIO, #A3007, USA) was orally treated at 50 mg kg body weight per day (mg/kg/d) for 7 days per cycle for two cycles, with a 2-week interval between cycles. Five cycles of Dasatinib (5 mg/kg/d; Sigma, #D33072G, USA) and Quercetin (50 mg/kg/d; Sigma, #Q4951, USA) were administered by oral gavage, with a week in between each cycle. The dosage of two senolytic drugs for oral gavage was determined from previous studies showing ABT263 [38] and DQ [48] decreased the senescence burden in various tissues. All mice were randomly assigned to the vehicle, ABT263, or D+Q treatment groups.

### Pain behavior test

### Von Frey filaments test

Von Frey filaments test was performed to detect mechanical allodynia with von Frey filaments (Stoelting Co., #58011, USA) days 2 and 14 after the last drug dosage. Mice were placed in a plastic chamber (60 X 100 X 60 mm) to habituate 30 mins for three days before the test. After acclimation, the mechanical withdrawal threshold of the hind paw was measured with von Frey filaments (0.02, 0.07, 0.16, 0.40, 0.60, 1.0, 2.0, and 4.0 in grams) applied on the plantar surface of the hind paws over a 10 mins interval for at least six separate measurements. The withdrawal threshold of the hind paw was calculated by the up-and-down method of Dixon and Mood [25]. A 0.4 g filament was first applied to judge the development of neuropathic pain. The next smaller filament was used if an avoidance response occurred, and the next higher one was used if no withdrawal response was observed.

### Hargreaves test

Pain behavior was evaluated with von Frey filaments as described previously [24]. To measure thermal hyperalgesia, mice were placed in a plantar analgesia meter plastic chamber (San Diego Instruments, #390G, USA). Halogen light was positioned at the hind paw. The time for avoidance response was recorded as a withdrawal latency (s). At least three measurements were taken per mouse over 10 min. A cut-off time was set by 20 secs to prevent injury.

### Grip test analysis

Grip strength was performed three days after drug administration. We placed the mice on the grip strength test apparatus (Biosebs, #BIO-GS3, USA) and waited until they grabbed the metal grid. The mice were pulled to record the force acquired from the apparatus. The peak of the graph was recorded, and we averaged 10 trials.

### Knee joint and DRG collection

Mice were anesthetized with 3% isoflurane 14 days after the last drug treatment. Dissected knee joints were transferred to liquid nitrogen, snap-frozen, and stored at -80° C to extract RNA or bathed in 10% formalin for histological assessment of the medial tibial plateau joint after the muscles around the joints were removed by scissors. To isolate ipsilateral L4 DRG, the skin on the spine was cut and the soft tissue was dissected from the spinous processes to expose the spinal vertebrae. The vertebral arch from the spinal cord was detached, avoiding spinal cord damage. L4 DRG dissected from the spinal cord was either dehydrated by 20% sucrose in PBS overnight at 4° C and embedded in OCT (Tissue-Tek®, Sakura Finetek, Japan) for immunostaining or flash-frozen using liquid nitrogen for RNA isolation.

### Histology

Mouse knee joints were fixed in 10% formalin overnight and decalcified using 10% ethylenediaminetetraacetic acid (EDTA) in PBS (PH 7.4) for 14 days. Decalcified joints were dehydrated using ethanol (50%, 70%, 80%, 95%, and 100%) and xylene for 10 min and embedded in paraffin. Sections (7 μm) were cut from the paraffin blocks in the sagittal orientation. The slides were stained in 0.05% Fast Green solution (Biosesang, #FR1001-005-02, Korea) for 5 min and dipped in 1% acetic acid 10 times. The sections were stained for proteoglycans with 0.1% Safranin-O (Biosesang, #SR1037-025-00, Korea) in distilled water for 25 min. The specimens were dehydrated using ethanol and submerged in xylene. The sections were mounted by Fisher Chemical™ Permount™ Mounting Medium (Fisher, #SP15-100, USA).

### Immunohistochemistry

For immunohistochemical analysis, paraffin-embedded knee joints on glass slides were deparaffinized by xylene and hydrated through a graded series of ethanol (100%, 95%, 80%, and 70%). Slides were incubated with 10 mM citrate buffer (pH 7; Biosesang, SR2189-050-60, Korea), heated in a microwave for 5 mins, and cooled to room temperature (RT) for 20 mins for antigen retrieval. The sections were then quenched with 0.3% H_2_O_2_ in methanol for 15 mins and blocked with 5% normal goat serum containing 0.3 % (v/v) Triton X-100 in phosphate-buffered saline (PBS) for 1 hr at RT. The VECTASTAIN Elite ABC HRP kit (Vector Laboratories, PK-6106, USA) was used per the manufacturer’s protocol. Primary antibodies against p16^INK4a^ (1:250, Abcam, #ab54210, USA) and HMGB1 (1:1000, Abcam, #ab18256, USA) were incubated overnight at 4° C, followed by incubation with a DAB substrate (Vector Laboratories, #SK-4100, USA). The sections were counterstained with Hematoxylin Solution (Mayer's, Modified) (Abcam, #ab220365, USA) for 10 min. The sections were dehydrated with ascending grades of ethanol and submerged in xylene. The stained slides were mounted with Fisher Chemical™ Permount™ Mounting Medium (Fisher, #SP15-100, USA). The percentage of p16+ or HMGB1+ cells were normalized to the total number of chondrocytes or synovial cells in the articular cartilage or synovium image taken at 40X (~ 130 mm^2^) using Image J 1.8.0 (NIH, USA).

### Pathological assessment of the knee joint

To assess OA’s histological changes, we quantitatively evaluated the articular cartilage of the tibia on Safranin-O-stained joint slides using the Osteoarthritis Research Society International (OARSI) scoring system. The OARSI grade consisting of six grades was used for semi-quantitative evaluation of cartilage degradation severity (surface intact, vertical fissures, erosion, degradation, and deformation) [22]. For quantitative evaluation of synovial inflammation, we used the Krenn-synovitis scoring system [23]. Grading the synovial membrane was carried out on Safranin-O-stained joint slides based on three synovial membrane features (surficial lining cell layer, density of resident cells, and cell infiltration). Each was judged on a three-level scale and each score was summed and shown as a Krenn-synovitis score. Osteophyte thickness (the distance on the marginal zone of the medial tibial plateau starting at the original edge of the tibial articular cartilage) was measured with at least three sections of Safranin-O-stained joint slides by image J software (NIH, USA).

### Histomorphometrical analyses for articular cartilage

All histomorphometrical quantifications were conducted from Safranin-O staining images as described above. The cartilage thickness and area were calculated as an average of three tibial articular cartilage length measurements in each slide. The calcified cartilage was measured in the thin inter-layer in each cartilage zone. Subchondral bone thickness and area were measured between the cartilage and growth plate in the tibial articular cartilage. Subchondral bone thickness and area were acquired by calculating an average of three measurements using Image J (NIH, USA).

### Immunofluorescence

Histological preparations for L4 DRGs sections (10 μm) were performed as follows. The first step was hydration and removal of OCT in distilled water for 3 min, and then sections were blocked with 5% normal goat serum containing 0.3% Triton X-100 in PBS for 1 hr at RT. Knee joint and L4 DRG sections were subsequently incubated with primary antibodies against CGRP (1:200, Abcam, #ab81887), TrkA (1:200, Millipore, #06-574), NGF (1:200, Abcam, #ab6199) and CD31 (1:500, Abcam, #ab124432) in blocking solution overnight at 4° C, washed three times for 5 min each in PBS, incubated with a secondary antibody conjugated with fluorescence (1:200, Invitrogen, #A11032 or #A11012, USA) for 1 hr at RT, stained with DAPI (Merck, #D9542, USA), and mounted with Fluoromount™ Aqueous Mounting Medium (Sigma, #F4680, USA). Stained slides underwent fluorescence microscopy (Olympus, #DP71, Japan). CGRP and TrkA positive neuro-fiber densities in the synovium were measured by calculating the positive area divided by the total synovial membrane area (mm^2^/ mm^2^). The percentage of the NGF and area was analyzed as the positive area of the NGF/total synovium membrane area. CD31 positive-blood vessels area was quantified as a percentage of the entire 40X synovial membrane area using Image J (NIH, USA).

### TRAP staining

Paraffin-embedded joint slides were left on a hot plate at 55° C for 10 mins, deparaffinized by xylene, and hydrated through a graded ethanol series. The sections were stained for TRAP-positive cells using the K-assay Trap staining kit (Kamiya-Biomedical Company, #KT-008, USA) per the manufacturer’s instruction. After staining, sections were washed by distilled water, stained with hematoxylin (Abcam, USA), dehydrated through ascending ethanol grades, submerged in xylene for 1 min, and mounted with Fisher Chemical™ Permount™ Mounting Medium (Fisher, #SP15-100, USA). Bone marrow images in the subchondral bone were obtained by light microscopy (Olympus, DP71). The percentage of the TRAP-positive area was then measured using Image J (NIH, USA). For quantification, the percentage of the positively stained area was divided by the total subchondral bone marrow area using Image J (NIH, USA).

### Human synoviocyte isolation and culture

Human synovial membrane samples were obtained from patients with OA undergoing total knee replacement arthroplasty surgery according to the study protocol approved by the Korea University of College of Medicine Institutional Review Board (IRB-2020-0223). Synovial membranes were washed three times with high-glucose DMEM (Corning, #10-090-CV, USA) and supplemented with 100 U/ml of penicillin and 100 ug/ml of streptomycin (P/S; Gibco, #15140122, USA). The synovial membrane was cut into 1 mm^3^ pieces and digested on the shaker for 3 hrs at 37° C in a CO_2_ incubator with Dulbecco's modified eagle's media (DMEM; Corning, #10-090-CV, USA) containing 1 mg/ml of collagenase type 1 (Worthington, #LS004196, USA). Digested synovia was centrifuged at 1,000 rpm for 10 mins. The supernatant was discarded. The solution was added to 0.25% trypsin-EDTA (Gibco, #25200056, USA) in a CO_2_ incubator. After 1 hr of digestion, the filtrate was passed through a 70 μm strainer. Cells were then washed twice in PBS and cultured in DMEM supplemented with 10% FBS (Gibco, #16140071, USA) and 1% P/S (growth media). Growth media were changed three times weekly. We used synoviocytes at passage three to six to remove potential immune cells.

For SA-β-gal from synoviocytes isolated from OA patients, 1,600 cells were seeded in a 96-well cell culture plate. For Tunel staining, 8,500 cells were seeded in 4 well chamber slides (Thermo Scientific, #154526, USA). Synoviocytes were maintained at 37° C with 5% CO_2_ in synoviocyte growth medium. One day after cell seeding, the cells were treated with ABT263 (2.5 μM, 1.25 μM, and 0.625 μM) for three days or D+Q (Dasatinib at 1,000 nM, 500 nM, and 250 nM plus Quercetin at 200 μM, 100 μM, and 50 μM) for one day, or the vehicle (DMSO). At the treatment endpoint, we conducted SA-β-gal and Tunel staining. To isolate RNA, 40,000 cells were seeded in a 6 well plate and incubated at 37° C with 5% CO_2_ in a growth medium. After one day, 1.25 μM of ABT263 in growth media was added for three days. D+Q (Dasatinib at 500 nM + Quercetin at 100 μM) was added to the growth media for one day. The senolytic-treated media was discarded and the growth media was changed three times per week. RNA isolation was conducted 10 days after drug treatment.

### TUNEL staining

Synoviocytes from OA human patients were seeded in a 4 well chamber slide for tunnel staining as described above. The DeadEnd™ Fluorometric TUNEL System, tunnel staining kit (Promega, #G3250, USA) was used for staining per the manufacturer’s instruction. DAPI was added for counterstaining. Stained cells were mounted with Fluoromount™ Aqueous Mounting Medium (Sigma, #F4680, USA). Tunnel staining images were acquired with fluorescence microscopy (Olympus, #DP71, Japan). Quantification tunnel positive cells were divided by total cells (DAPI positive cells) by Image J (NIH, USA).

### SA-β-gal staining

SA-β-gal staining was performed using a Senescence β-Galactosidase Staining Kit (Cell Signaling Technology, #9860, USA) per the manufacturer’s instructions. SA-β-gal-positive senescent cells were identified as blue-stained cells under light microscopy. For the cell culture experiments, cells were counted using nuclear DAPI counterstain in 8 random fields per culture dish to determine the percentage of SA-β-gal-positive cells by using EVOS M5000 (Thermo Fisher Scientific, USA). SA-β-gal positive cells were analyzed by dividing the total cells with Image J (NIH, USA).

### RNA isolation and real-time

The knee joints were stored at -80° C and immediately transferred to liquid nitrogen and homogenized with a mortar and pestle. Total RNA of knee joints and synoviocytes from OA patients was isolated using TRIzol™ Reagent (Invitrogen, #15596018, USA) and RNA mini-prep kit (Zymo-Research, #R2052, USA) per the manufacturer’s protocol. The concentration of the extracted RNA was evaluated using nanodrop (Thermo Scientific, #ND-2000C, USA). Total RNA was reverse transcribed to cDNA using a High-Capacity cDNA Reverse Transcription kit (Thermo Scientific, #4368814, USA) per the manufacturer’s instructions. Real-time PCR was performed using a Quantstudio 3 Real-Time PCR system (Applied Biosystems, USA) with SYBR™ Green PCR Master Mix (Thermo Scientific, USA). All signals were normalized to that of *β-actin*. Relative mRNA levels were calculated using the ^ΔΔ^C_T_ analysis with the mean C_T_ values of the collective endogenous controls for internal normalization. The primer sequences are listed in Supplementary Table 2.

### Statistics

All data were analyzed by GraphPad Prism 7.0. The unpaired Student’s t-test was used to compare statistical significance. We assumed significance when p < 0.05.

## Supplementary Material

Supplementary Figures

Supplementary Tables
