# Supplementary material for: Senolytic drugs relieve pain by reducing peripheral nociceptive signaling without modifying joint tissue damage in spontaneous osteoarthritis
Source: Aging (Albany NY). 2022 Aug 10;14(15):6006–27. doi: 10.18632/aging.204204 (PMC9417227; doi:10.18632/aging.204204)
Supplement: Supplementary Figures [file aging-14-204204-s001.pdf]

SUPPLEMENTARY FIGURES

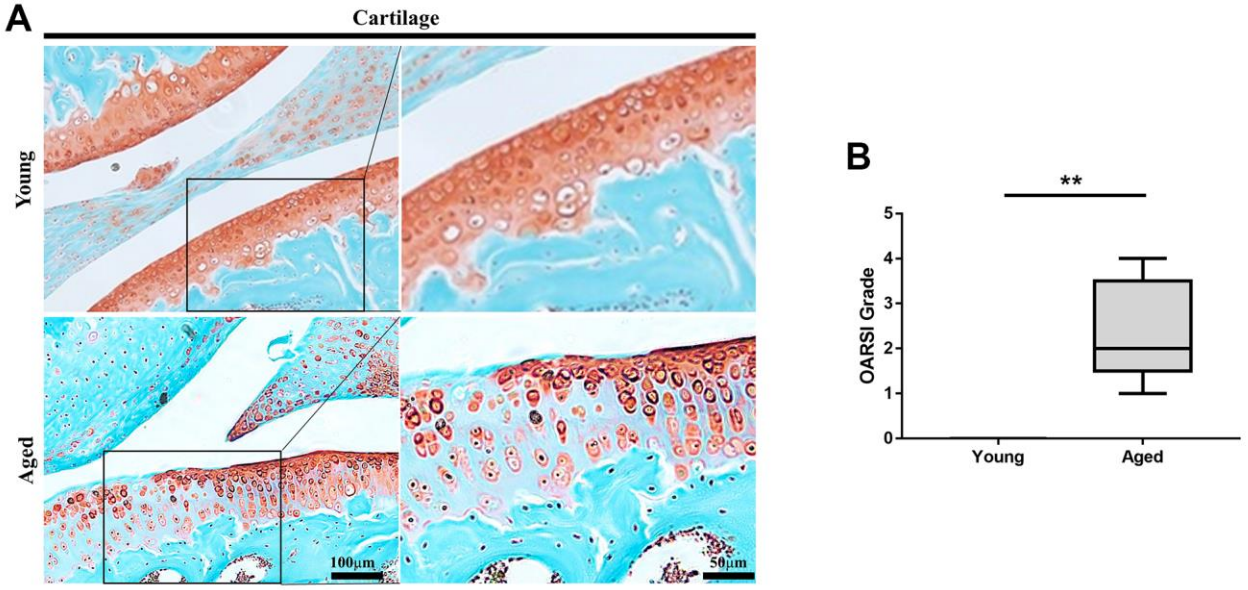

Supplementary Figure 1. Histological characterization of *in vivo* mouse spontaneous osteoarthritis model. (**A**) Representative Safranin-O stained sections of the articular joints of 10-weeks (Young) and 20 to 21-month (Aged)-old mice (n = 5 for Young, n = 5 for Aged). Right panels show a higher magnification of the boxed regions indicated in the left panels. (**B**). OARSI grades for articular joints as shown in A. Whisker plots represent the 10 and 90 percentiles, and the lines correspond to the median. ** p < 0.01; two-tailed Student's t-test (unpaired). Scale bars are shown in each image.

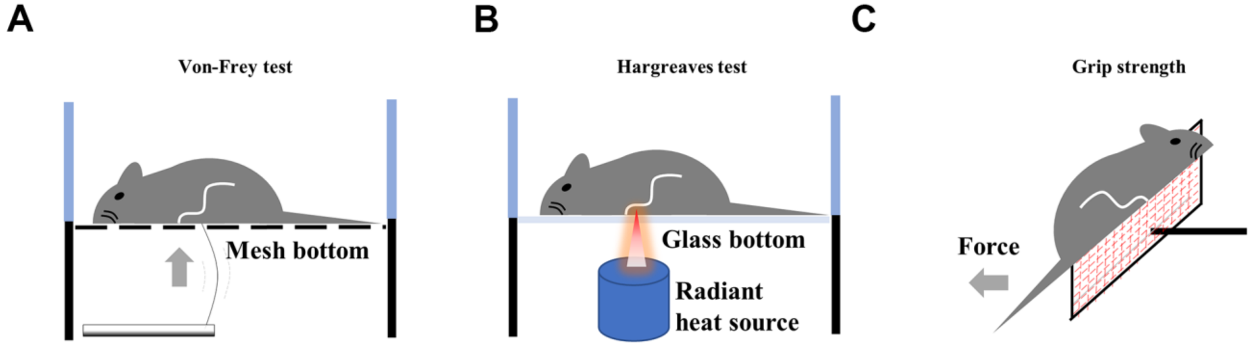

Supplementary Figure 2. The schematic diagram of the apparatus used in performing pain-associated behavioral experiment. (**A**) Von-Frey test, (**B**) Hargreaves test, and (**C**) Grip strength.

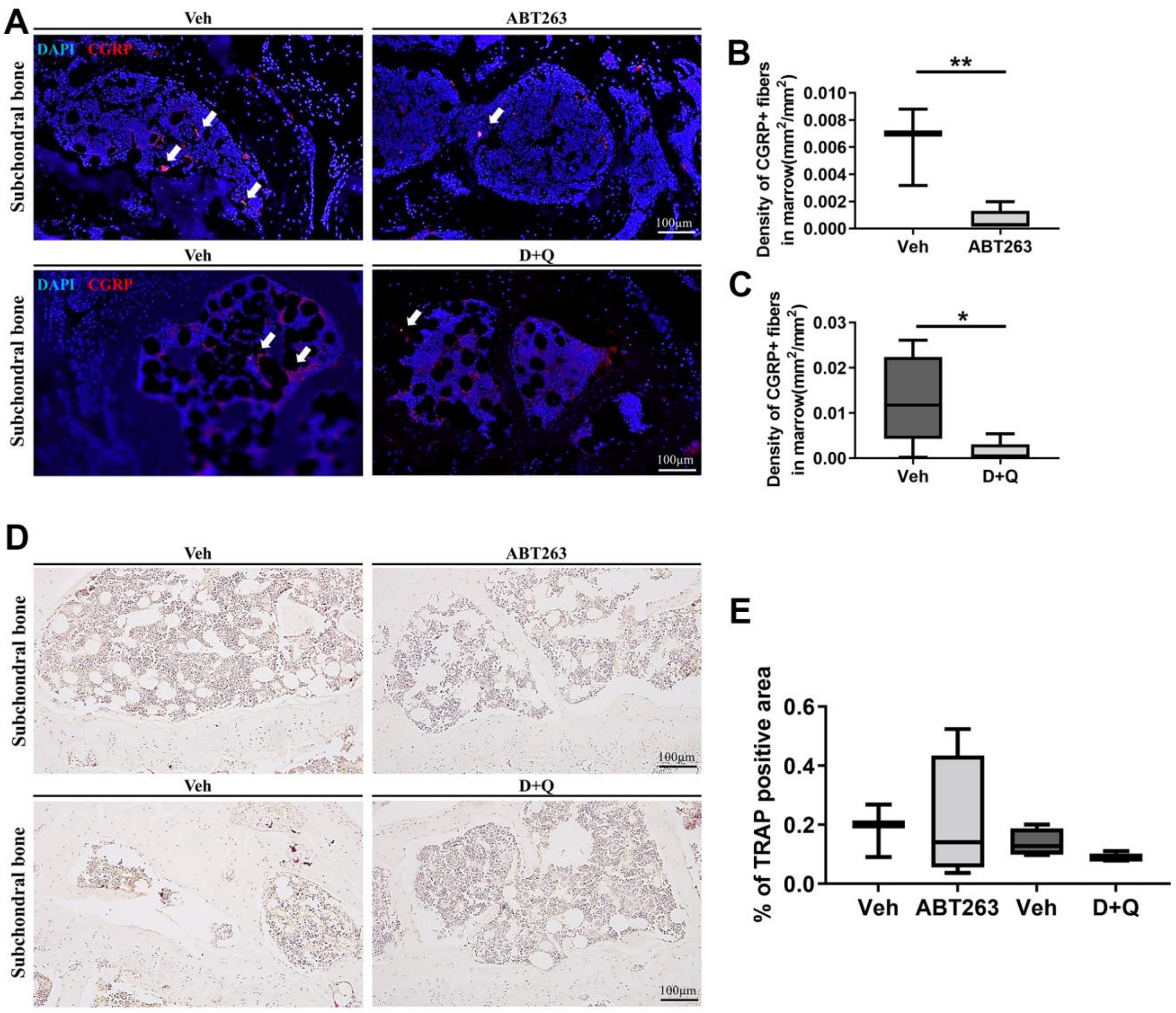

Supplementary Figure 3. Sprouting of CGRP+ sensory nerves in subchondral bone was decreased by ABT263 and D+Q treatment but osteoclast activity was not affected by those senolytic agents. (**A**) Representative immunofluorescence images for CGRP staining in subchondral bone of 21 to 22-month-old mice treated with ABT263 or D+Q. Density of CGRP+ nerve fibers in subchondral bone marrow of (**B**) ABT263 (n = 3 for Veh, n = 5 for ABT263) or (**C**) D+Q treated aged mice joints (n = 5 per group). (**D**) Representative images of TRAP staining and (**E**) quantitative analysis of TRAP + area in subchondral bone of ABT263 (n = 3 for Veh, n = 4 for ABT263) or D+Q-treated aged mice (n = 4 for Veh, n = 3 for D+Q). Whisker plots represent the 10 and 90 percentiles, and the line corresponds to the median. * $p < 0.05$, ** $p < 0.01$; Unpaired Student's t-test. Scale bars, 100 μm.

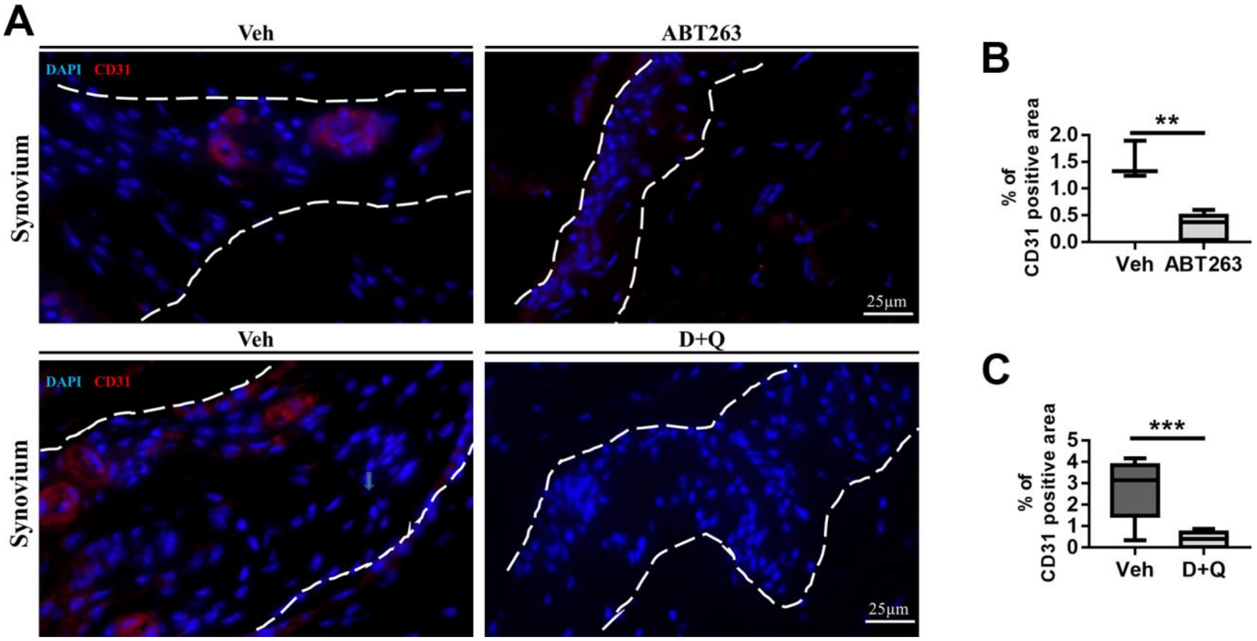

Supplementary Figure 4. ABT263 and D+Q treatment attenuated synovial vasculature developed in the spontaneous OA mouse model. (**A**) Representative images for CD31 immunofluorescence staining in the synovium of 21 to 22-month-old mice treated with ABT263 or D+Q (n = 3 for Veh, n = 5 for ABT263 and n = 5 for Veh, n = 8 for D+Q). Dashed lines indicated a synovial membrane region. Quantification of CD31+ area as a percentage of the entire 40X area in the synovium of (**B**) ABT263 or (**C**) D+Q group. Whisker plots represent the 10 and 90 percentiles, and the lines correspond to the median. ** $p < 0.01$, *** $p < 0.001$; Unpaired Student's t-test. Scale bars, 25 μm.
