# Supplementary material for: Senolytic drugs relieve pain by reducing peripheral nociceptive signaling without modifying joint tissue damage in spontaneous osteoarthritis
Source: Aging (Albany NY). 2022 Aug 10;14(15):6006–27. doi: 10.18632/aging.204204 (PMC9417227; doi:10.18632/aging.204204)
Supplement: Supplementary Tables [file aging-14-204204-s002.pdf]

SUPPLEMENTARY TABLES

Supplementary Table 1. Histomorphometric measurements of joint articular cartilage thickness and area.

	Veh	ABT263	p-value	Veh	D+Q	p-value
	Average ± SD			Average ± SD		
**Cartilage**						
Thickness (μm)	71.87 ± 5.03	71.39 ± 9.00	n.s	78.22 ± 8.55	73.74 ± 15.57	n.s
Area (mm^2)	151.24 ± 27.94	131.04 ± 22.60	n.s	149.09 ± 9.91	124.56 ± 40.08	n.s
**Calcified Cartilage**						
Thickness (μm)	47.40 ± 5.34	43.21 ± 5.29	n.s	41.98 ± 5.73	49.59 ± 10.29	n.s
Area (mm^2)	62.26 ± 19.60	58.03 ± 7.96	n.s	65.85 ± 10.87	67.22 ± 25.51	n.s
**Subchondral bone**						
Thickness (μm)	59.95 ± 13.98	51.95 ± 11.97	n.s	64.39 ± 22.74	65.01 ± 26.75	n.s
Area (mm^2)	74.72 ± 18.62	70.47 ± 18.26	n.s	100.14 ± 49.09	88.52 ± 40.05	n.s

*SD, standard deviation.
*n.s, not significant.

Supplementary Table 2. Primer sequences of qRT-PCR.

Gene symbol	Primer sequence (5′-3′)
Ngf (mouse)	Forward, ACTGGACTAAACTTCAGCATTCC<br>Reverse, GGGCAGCTATTGGTGCAGTA
Cdkn2a (mouse)	Forward, AATCTCCGCGAGGAAAGC<br>Reverse, GTCTGCAGCGGACTCCAT
IL-1β (mouse)	Forward, GTATGGGCTGGACTGTTTC<br>Reverse, GCTGTCTGCTCATTCACG
Atf4 (mouse)	Forward, ATGGCGCTCTTCACGAAATC<br>Reverse, ACTGGTCGAAGGGGTCATCAA
Vegf (mouse)	Forward, CTGCCGTCCGATTGAGACC<br>Reverse, CCCCTCCTTGTACCACTGTC
IL-6 (mouse)	Forward, GCTACCAAACTGGATATAATCAGG<br>Reverse, CCAGGTAGCTATGGTACTCCAGAA
β-actin (mouse)	Forward, CAACCGTGAAAAGATGACCC<br>Reverse, GTAGATGGGCACAGTGTGGG
NGF (human)	Forward, GGCAGACCCGCAACATTACT<br>Reverse, CACCACCGACCTCGAAGTC
CDKN2A (human)	Forward, CCAACGCACCGAATAGTTACG<br>Reverse, GCGCTGCCCATCATCATG
CDKN1A (human)	Forward, TGTCCGTCAGAACCCATGC<br>Reverse, AAAGTCGAAGTTCCATCGCTC
ATF4 (human)	Forward, CCCTTCACCTTCTTACAACCTC<br>Reverse, TGCCCAGCTCTAAACTAAAGGA
VEGF (human)	Forward, ATGACCGAAATGAGCTTCCTG<br>Reverse, GCTGGAGAACCCATGAGGT
IL-6 (human)	Forward, CCCCTGACCCAACCACAAAT<br>Reverse, ATTTGCCGAAGAGCCCTCAG
β-ACTIN (human)	Forward, GCTCCTCCTGAG<br>Reverse, GTAGATGGGCACAGTGTGGG
